# Tofacitinib does not affect platelet function or coagulation in patients with ulcerative colitis

**DOI:** 10.3389/fimmu.2026.1941163

**Published:** 2026-09-10

**Authors:** Fabio Suarez-Trujillo, Paula J. Martínez, Irene Soleto, Mar Meijón, Ana Kerguelen, Mar Orts, Antonio Planas, Natalia Acedo, Guillermo Chacón-Calvo, Ana Garre, Elena Moreno-Lopez, Nora Butta, María Chaparro, Montse Baldán-Martín, Javier P. Gisbert

**Affiliations:** 1 Inflammatory Bowel Disease Unit, Hospital Universitario de La Princesa, Instituto de Investigation Sanitaria Princesa (IIS-Princesa), Universidad Autónoma de Madrid (UAM), Centro de Investigación Biomédica en Red de Enfermedades Hepáticas y Digestivas (CIBEREHD), Madrid, Spain; 2 Hematology and Hemotherapy Service, Thrombosis and Haemostasis laboratory, Hospital Universitario Ramón y Cajal, Instituto Ramón y Cajal de Investigación Sanitaria (IRYCIS), Madrid, Spain; 3 Hematology Service, Hospital Universitario de La Paz-IdiPAZ, Madrid, Spain; 4 Anesthesiology Service, Hospital Universitario de La Princesa, Madrid, Spain; 5 Hematology Service, Hospital Universitario de La Princesa, Madrid, Spain; 6 Coagulopathies and Hemostasis Disorders Group, Hospital Universitario de La Paz-IdiPAZ, Madrid, Spain

**Keywords:** hemostasis, JAK inhibitors, platelet function, thrombosis, tofacitinib, ulcerative colitis

## Abstract

**Background:**

Tofacitinib is a Janus kinase inhibitor used to treat inflammatory diseases, including rheumatoid arthritis and ulcerative colitis (UC). Previous studies have reported an increased risk of thromboembolic events in patients with rheumatoid arthritis treated with tofacitinib. Therefore, evaluating whether these effects are also present in other inflammatory diseases such as UC is important.

**Methods:**

In this exploratory study, we evaluated the impact of tofacitinib on platelet function and coagulation profiles in patients with active UC receiving tofacitinib (n=10), compared with patients treated with anti-TNF agents (n=10) and healthy controls (HC; n=10). Patients were assessed at baseline and after 14 weeks of treatment. Platelet aggregation was assessed by lumiaggregometry, while platelet activation was evaluated by flow cytometry through PAC-1 binding and surface expression of CD62P and CD63. Coagulation was assessed by rotational thromboelastometry (ROTEM^®^) using extrinsic (EXTEM) and intrinsic (INTEM) pathway activation assays. Longitudinal analyses evaluated the effects of time, treatment, and their interaction.

**Results:**

Tofacitinib did not significantly affect platelet aggregation or the expression of platelet activation markers compared with either anti-TNF-treated patients or HC. Regarding thrombus dynamics, a significant difference was observed only in the INTEM assay when comparing the tofacitinib and anti-TNF groups, showing longer clot formation times in tofacitinib-treated patients; however, neither group differed from HC.

**Conclusions:**

In this study, tofacitinib treatment was not associated with increased platelet aggregation, platelet activation, or enhanced coagulation in patients with UC.

## 1 Introduction

Ulcerative colitis (UC) is a chronic immune-mediated inflammatory disease characterized by relapsing inflammation of the colonic mucosa (1, 2). Although therapeutic options have expanded substantially over recent decades, UC remains incurable, and many patients require long-term immunomodulatory treatment to achieve and maintain disease control. The introduction of advanced therapies, including biologics and small molecules, has transformed the management of UC, particularly in patients with moderate-to-severe disease or inadequate response to conventional agents (3–5).

Tofacitinib is an oral Janus kinase inhibitor approved for the treatment of moderate-to-severe UC. By inhibiting Janus kinase-dependent intracellular signaling pathways, tofacitinib interferes with multiple cytokine-mediated inflammatory responses involved in UC pathogenesis. Its rapid onset of action and oral administration have made it a valuable therapeutic option, especially in patients with refractory disease or previous exposure to biologic therapies (6, 7).

However, concerns have emerged regarding the thromboembolic and cardiovascular safety profile of tofacitinib. In patients with rheumatoid arthritis, particularly those older than 50 years and with cardiovascular risk factors, tofacitinib was associated with a higher incidence of venous thromboembolism, pulmonary embolism, and major adverse cardiovascular events compared with anti-TNF therapy (8). These findings led regulatory agencies, including the European Medicines Agency and the United States Food and Drug Administration, to issue safety warnings and recommend caution when prescribing tofacitinib to patients at increased thrombotic risk (9, 10).

The relevance of these findings to patients with UC remains incompletely understood. This is particularly important because UC itself is associated with an increased risk of venous and arterial thrombotic events, especially during active inflammation (11). Whether the thrombotic signal observed with tofacitinib reflects patient-related risk factors, disease-related inflammation, or a direct drug-related effect on hemostasis remains unclear. Clarifying the mechanisms underlying this potential risk could help identify patients who might benefit from targeted preventive strategies, including thromboprophylaxis or other risk-adapted interventions. In this context, evaluating coagulation parameters and platelet function in patients with active UC treated with tofacitinib may provide mechanistic insight into its potential prothrombotic profile.

Therefore, the aim of the present study was to assess whether tofacitinib treatment induces changes in coagulation parameters or platelet function in patients with active UC, and to compare these effects with those observed in patients treated with anti-TNF therapy.

## 2 Methods

### 2.1 Study design and blood samples collection

A total of 30 participants were included in the study: 10 patients with UC receiving anti-TNF treatment (infliximab or adalimumab), 10 patients with UC receiving tofacitinib, and 10 healthy controls (HC). Blood samples from each patient were collected at the beginning of the treatment (baseline) and three months after (14-weeks visit). Given the exploratory mechanistic nature of the study, no formal *a priori* sample-size calculation based on a predefined clinically meaningful difference was performed. Therefore, the study was not designed as an equivalence or non-inferiority study or to exclude effects below a predefined magnitude. Estimated mean differences with 95% confidence intervals were reported alongside p-values for the relevant comparisons to provide information on the magnitude and precision of the observed effects.

Eligible participants were adults aged ≥18 years. Patients with UC were required to have an established diagnosis according to European Crohn’s and Colitis Organisation (ECCO) criteria. Concomitant therapies were permitted provided that they had remained stable for at least 3 months before study inclusion. UC had to have endoscopic activity, defined as a Mayo endoscopic subscore ≥2 documented within 1 month before treatment initiation. HC were individuals without UC or any other known inflammatory, allergic, malignant, or autoimmune disease. The study was approved by the Ethics Committee of Hospital Universitario de La Princesa (protocol code: GIS-2021-JAKihemo) based on the Declaration of Helsinki guidelines. All participating patients provided written informed consent, and all data were anonymized to ensure confidentiality.

Patients with UC were excluded if they had any concomitant immune-mediated disease, active infection, malignancy, pregnancy or breastfeeding, alcohol or drug abuse, ostomy, abdominal surgery within the previous 6 months, previous colectomy, active hepatitis B or C virus infection, human immunodeficiency virus infection, a history of thromboembolic events, hereditary coagulation disorders, current treatment with anticoagulants, antiplatelet agents, or other drugs affecting blood coagulation, or use of combined hormonal contraceptives or hormone replacement therapy.

Healthy controls were excluded if they had advanced chronic disease or any condition precluding compliance with the study protocol, pregnancy or breastfeeding, active hepatitis B or C virus infection, human immunodeficiency virus infection, alcohol or drug abuse, a history of thromboembolic events, hereditary coagulation disorders, current treatment with anticoagulants, antiplatelet agents, or other drugs affecting blood coagulation, or use of combined hormonal contraceptives or hormone replacement therapy.

### 2.2 Evaluation of platelet aggregation by lumiaggregometry

For platelet aggregation assays, whole blood was collected in presence of sodium citrate and immediately centrifuged at 900 rpm for 8 minutes at room temperature without brakes following the common clinical protocol to obtain platelet-rich plasma (PRP). Autologous platelet-poor plasma (PPP) was obtained from each patient by centrifugation of whole blood at 3250 rpm for 20 min at room temperature. PRP was used for measuring aggregation in a lumiaggregometer (Helena Biosciences Europe, Beaumont, TX, USA) and was adjusted with patient’s own PPP to a platelet concentration of 200–300 x10³ platelets/µL. PPP also served as the reference blank for equipment calibration. Adenosine diphosphate (ADP)was added as aggregation inductor at concentrations of 2.5 µM and 5 µM, and aggregation was measured for 10 min by light transmission. Results were expressed as percentage of maximal aggregation.

### 2.3 Evaluation of platelet activation state by flow cytometry

Whole blood was centrifuged at 300 g for 15 min at room temperature without brakes to obtain PRP. Platelet number was not measured for each patient and PRP was subsequently diluted 1:5 with wash buffer containing 137 mM NaCl, 2.7 mM KCl, 1 mM NaH2PO4, 20 mM HEPES, 0.1% glucose, and 0.1% bovine serum albumin (pH 7.4), following the standardized protocol. Platelets were stained with previously titrated anti-CD41 (HIP8, PE; 1/100), anti-CD62P (AK-4, FITC; 1/30), anti-CD63 (H5C6, FITC; 1/30), and PAC-1 (PAC1, FITC; 1/30) monoclonal antibodies for 20 min at 4°C. Platelets were identified according to forward and side scatter properties and CD41 expression and activation was evaluated at baseline and after stimulation with 10 M thrombin receptor-activating peptide (TRAP; Roche Diagnostics, Rotkreuz, Switzerland) for 20 min. Samples were acquired on a BD FACSCanto II flow cytometer (BD Biosciences), and data were analyzed using FlowJo software version 10.8 (Ashland, OR, USA). Expression of CD62P, CD63, and binding of PAC-1 was used as a marker of platelet activation and was reported as percentage of positive platelets (**Supplementary Figure 1**).

### 2.4 Evaluation of blood coagulation by rotational thromboelastometry

Whole blood coagulation was assessed using rotational thromboelastometry ROTEM^®^ (TEM Innovations GmbH, Munich, Germany) according to the manufacturer’s instructions. The extrinsic and intrinsic coagulation pathways were evaluated, and maximum clot firmness (MCF), which reflects the maximum tensile strength of the thrombus; clotting time (CT), defined as the time from start of measurement until a clot firmness amplitude of 2 mm is reached; and clot formation time (CFT) as the time until amplitude of 20 mm is reached were measured.

### 2.5 Statistical analysis

Descriptive statistics were employed to report characteristics of the study population, including age, sex, body mass index, and smoking status (smoker or non-smoker). These data were presented as mean ± standard deviation (SD) or number of individuals (n) and percentage. Given the exploratory nature of the study, no formal *a priori* sample-size calculation based on a predefined clinically meaningful difference was performed. Estimated mean differences and their corresponding 95% confidence intervals were reported alongside adjusted p-values for the relevant comparisons to provide information on the magnitude and precision of the observed differences.

Statistical analyses were performed using GraphPad Prism software (version 9.5.1; GraphPad Software Inc., San Diego, CA, USA). Longitudinal changes in platelet aggregation and ROTEM^®^ parameters were analyzed using two-way repeated-measures ANOVA, with time (baseline and week 14) as the within-subject factor and treatment group (anti-TNF and tofacitinib) as the between-subject factor. When repeated measurements were incomplete, mixed-effects models fitted by restricted maximum likelihood were used instead. The time-by-treatment interaction was used to assess whether longitudinal changes differed between treatment groups. Prespecified within-treatment comparisons between baseline and week 14 and between-treatment comparisons at each time point were performed using Šídák’s correction for multiple comparisons. Effect estimates are reported with 95% confidence intervals.

Healthy controls (HC), who were assessed at a single time point, were not included in the longitudinal models. Comparisons involving HC were performed separately using two-way ANOVA followed by Tukey’s multiple-comparisons test. Platelet activation parameters assessed by flow cytometry were also analyzed using two-way ANOVA followed by Tukey’s multiple-comparisons test.

Additional exploratory analyses were performed to assess potential associations of sex and age with hemostatic parameters. Differences in platelet activation and ROTEM^®^ parameters between male and female patients were assessed using two-tailed Mann–Whitney U tests. Associations between age and platelet activation and ROTEM^®^ parameters were evaluated using two-tailed Spearman rank correlations. For the age correlation analyses, p-values were adjusted for multiple comparisons using the Benjamini–Hochberg false discovery rate procedure. False discovery rate-adjusted p-values were calculated using Python (version 3.13.5). Normality was assessed using the Shapiro–Wilk test and inspection of Q–Q plots. A p-value <0.05 was considered statistically significant.

## 3 Results

### 3.1 Baseline clinical characteristics

A total of 30 individuals were included in the study. No significant differences were observed in baseline characteristics of the study population such as sex, smoking status, and body mass index (BMI) between the different study groups. Average age of HC was significantly higher (p=0.003) than that of tofacitinib or anti-TNF groups (**Table 1**).

**Table 1:** Clinical characteristics of patients included in the study.

Variable	HC	UC patients with anti-TNF	UC patients with tofacitinib	p-value
Age (years), mean (SD)	62.5 (7)	40.9 (19)	47.1 (16)	0.003**
Female, n (%)	6 (60)	4 (40)	4 (40)	0.92
Smokers, n (%)	3 (30)	0 (0)	0 (0)	0.59
BMI, mean (SD)	25.7 (4)	23 (8)	27.05 (8)	0.91

BMI, body mass index; HC, healthy control; SD, standard deviation; UC, ulcerative colitis. **p<0.01.

### 3.2 Effect of tofacitinib on platelet aggregation

**Figure 1:**
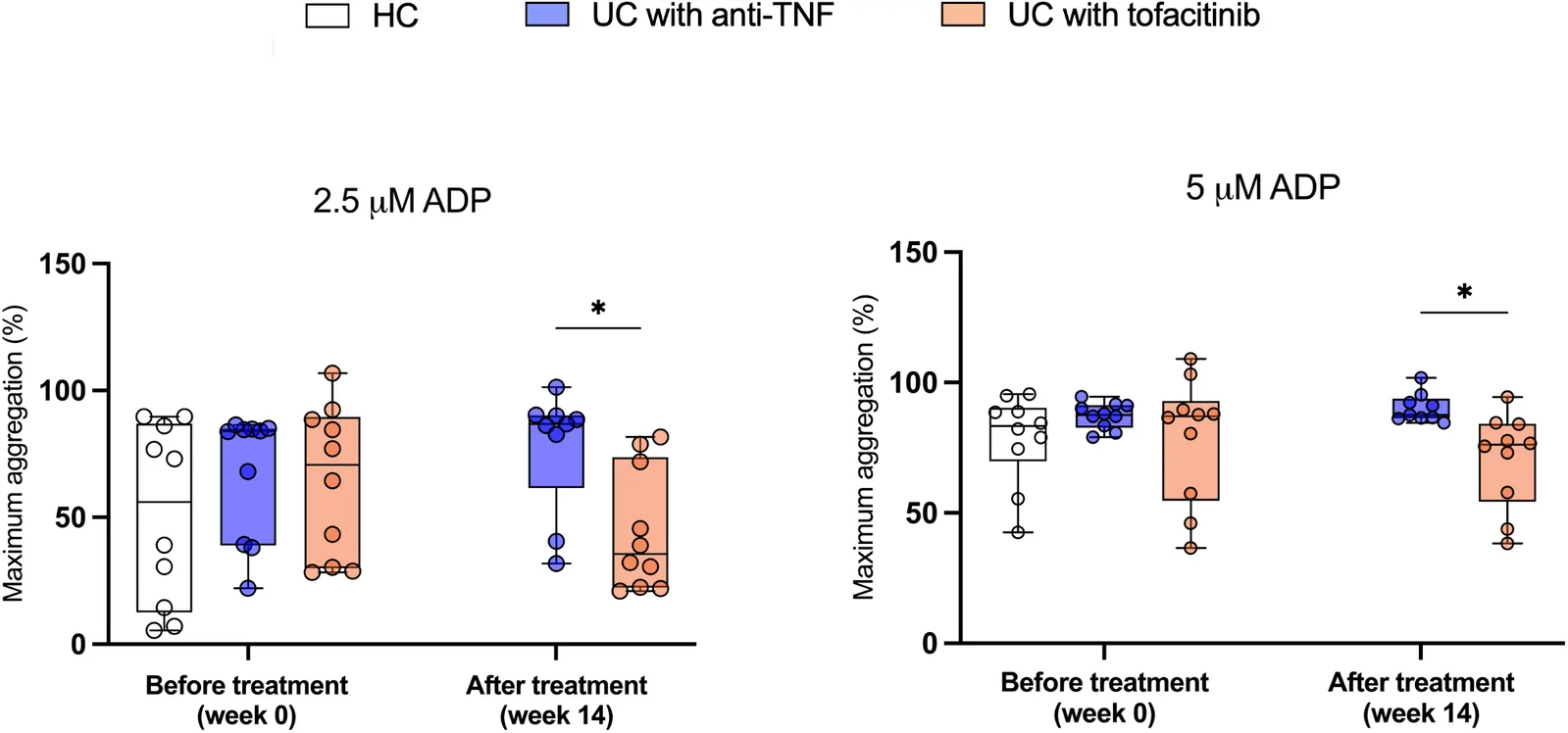
Platelet aggregation in patients with ulcerative colitis treated with anti-TNF or tofacitinib. Maximum platelet aggregation was measured in platelet-rich plasma obtained from healthy controls and patients with ulcerative colitis treated with anti-TNF or tofacitinib, using 2.5 or 5 µM adenosine diphosphate as aggregation inducer. Patients were assessed at baseline and after 14 weeks of treatment, whereas healthy controls were assessed at a single time point. Sample sizes varied according to data availability (n=9–10 per group). Longitudinal comparisons between anti-TNF- and tofacitinib-treated patients were performed using mixed-effects models, with time, treatment, and time × treatment as fixed effects. Prespecified within-treatment comparisons between baseline and week 14 and between-treatment comparisons at each time point were performed using Šídák’s multiple-comparisons test. Comparisons involving healthy controls were performed separately using two-way ANOVA followed by Tukey’s multiple-comparisons test. Data are presented as mean ± SD. *p<0.05. ADP, adenosine diphosphate; HC, healthy controls; SD, standard deviation; TNF, tumor necrosis factor; UC, ulcerative colitis.

Maximum platelet aggregation was measured in PRP following stimulation with 2.5 or 5 µM ADP (**Figure 1**). Longitudinal analysis showed a significant time-by-treatment interaction for aggregation induced by 2.5 µM ADP (p=0.045; 95% CI, −56.73 to −0.70), indicating different longitudinal trajectories between treatment groups. Although neither treatment group showed a statistically significant within-group change from baseline to week 14, aggregation at week 14 was significantly lower in patients receiving tofacitinib than in those receiving anti-TNF therapy (95% CI, 4.23 to 59.53; adjusted p=0.021), whereas no significant between-group difference was present at baseline (adjusted p=0.954) (**Figure 1**; **Supplementary Table 1**).

For aggregation induced by 5 µM ADP, no significant time-by-treatment interaction was observed (p=0.224; 95% CI, −28.11 to 7.07), although an overall treatment-group effect was detected (p=0.024). Aggregation did not change significantly from baseline to week 14 within either treatment group. While no significant difference between anti-TNF and tofacitinib was observed at baseline (95% CI, −7.51 to 25.22; adjusted p=0.383), aggregation was significantly lower in the tofacitinib group at week 14 (95% CI, 2.61 to 36.15; adjusted p=0.021) (**Figure 1**; **Supplementary Table 1**).

Comparisons with HC, who were assessed at a single time point, were performed separately as cross-sectional analyses, with no significant differences observed between HC and either treatment group.

### 3.3 Effect of tofacitinib on platelet activation

**Figure 2:**
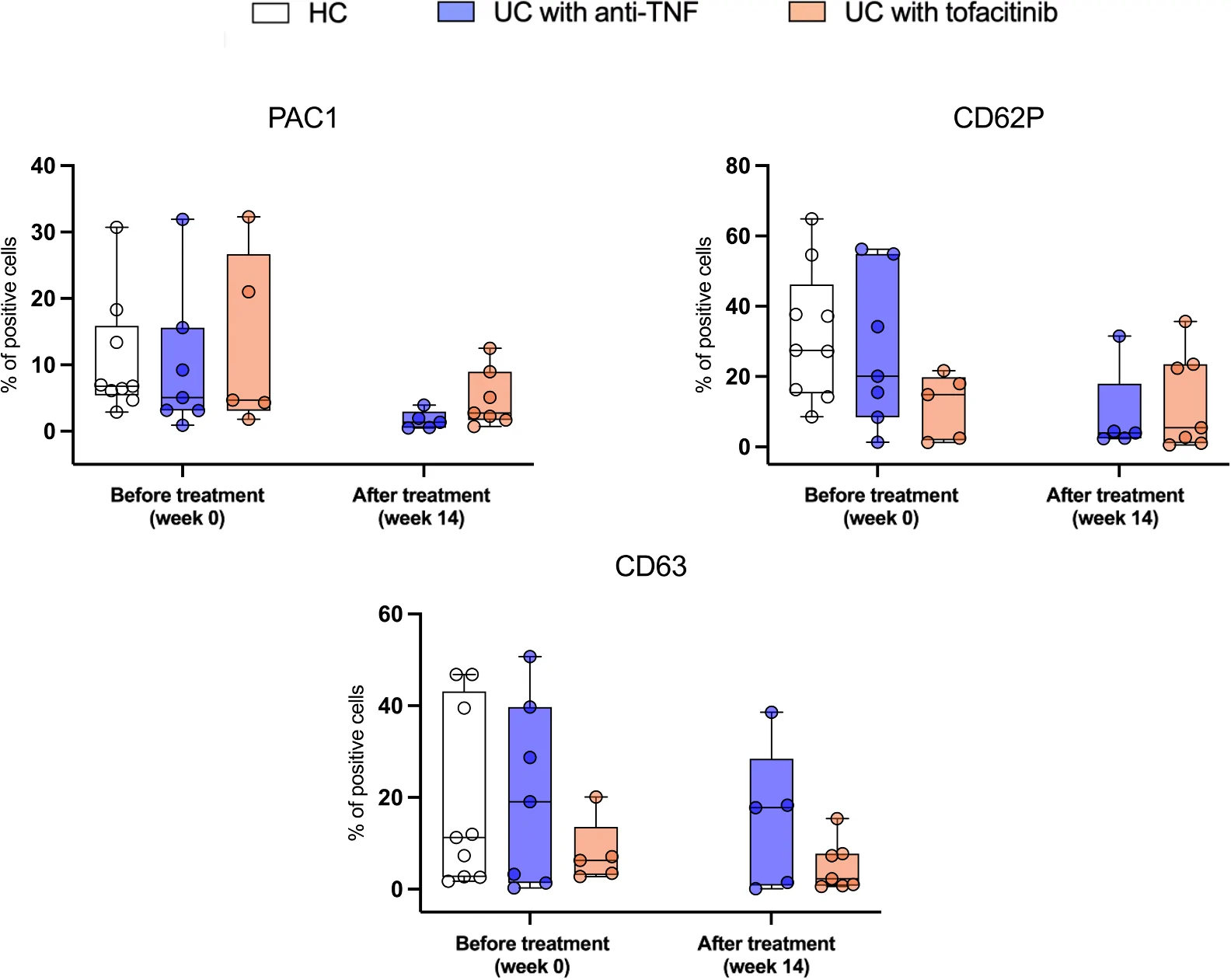
Platelet activation under unstimulated conditions in patients with ulcerative colitis treated with anti-TNF or tofacitinib. Platelet activation was assessed by flow cytometry through PAC-1 binding and surface expression of CD62P and CD63 in platelet-rich plasma obtained from healthy controls and patients with ulcerative colitis treated with anti-TNF or tofacitinib. Patients were assessed at baseline and after 14 weeks of treatment, whereas healthy controls were assessed at a single time point. Sample sizes varied according to data availability (n=5–9 per group). Comparisons were performed using two-way ANOVA followed by Tukey’s multiple-comparisons test. Data are presented as mean ± SD. CD, cluster of differentiation; HC, healthy controls; SD, standard deviation; TNF, tumor necrosis factor; UC, ulcerative colitis.

**Figure 3:**
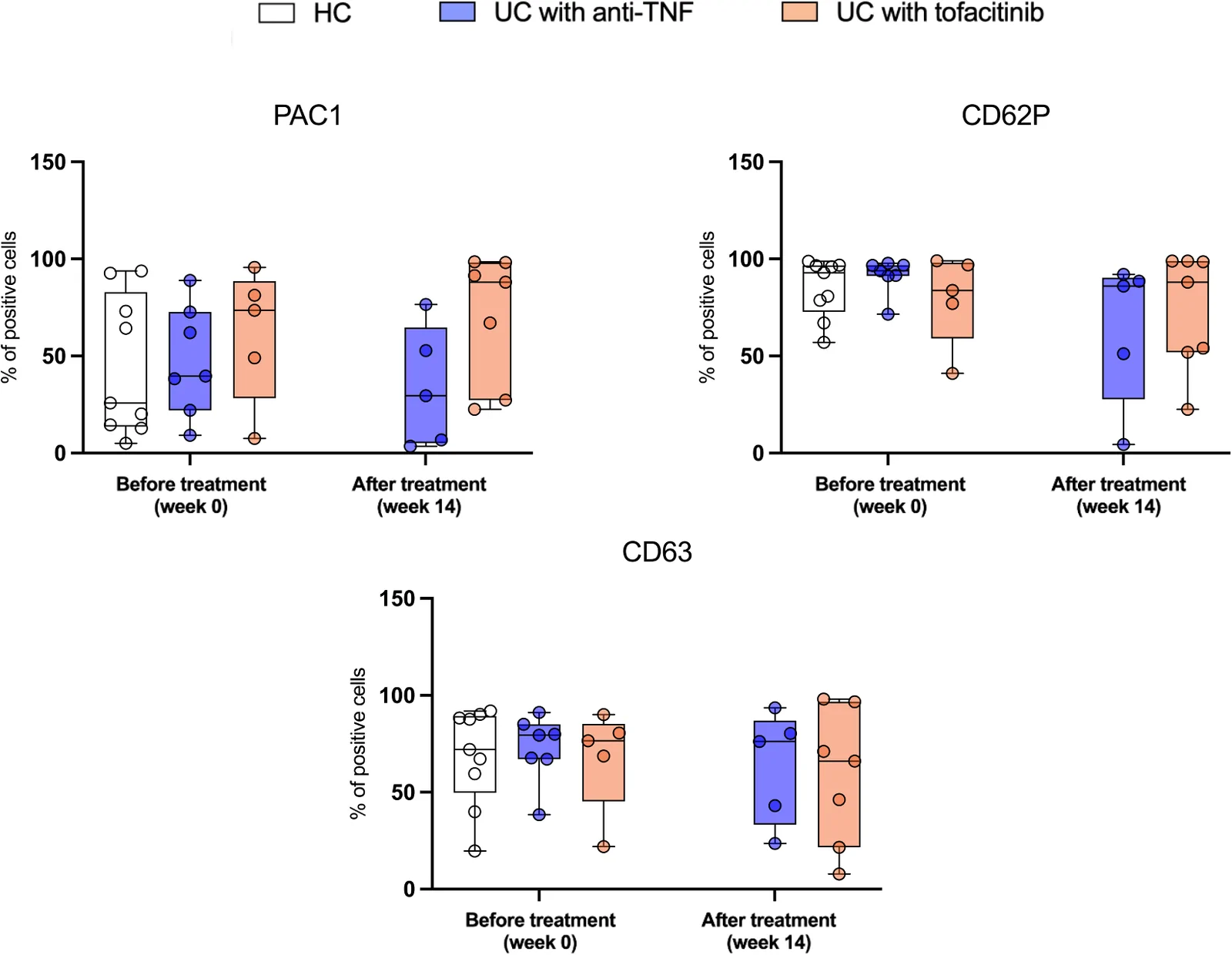
Platelet activation after thrombin receptor-activating peptide stimulation in patients with ulcerative colitis treated with anti-TNF or tofacitinib. Platelet activation was assessed by flow cytometry through PAC-1 binding and surface expression of CD62P and CD63 in platelet-rich plasma obtained from healthy controls and patients with ulcerative colitis treated with anti-TNF or tofacitinib after stimulation with thrombin receptor-activating peptide. Patients were assessed at baseline and after 14 weeks of treatment, whereas healthy controls were assessed at a single time point. Sample sizes varied according to data availability (n=5–9 per group). Comparisons were performed using two-way ANOVA followed by Tukey’s multiple-comparisons test. Data are presented as mean ± SD. CD, cluster of differentiation; HC, healthy controls; SD, standard deviation; TNF, tumor necrosis factor; UC, ulcerative colitis.

Platelet activation markers were detected by flow cytometry in PRP samples obtained from patients with UC treated with tofacitinib, anti-TNF and HC. As shown in **Figure 2**, tofacitinib did not increase either the binding of PAC-1, nor the expression CD62P (P-selectin) and CD63 activation markers in comparison with anti-TNF or no treatment. Similar findings were observed after stimulation with TRAP, with no significant differences in activation marker expression among groups (**Figure 3**).

### 3.4 Effect of tofacitinib on thrombus dynamics

**Figure 4:**
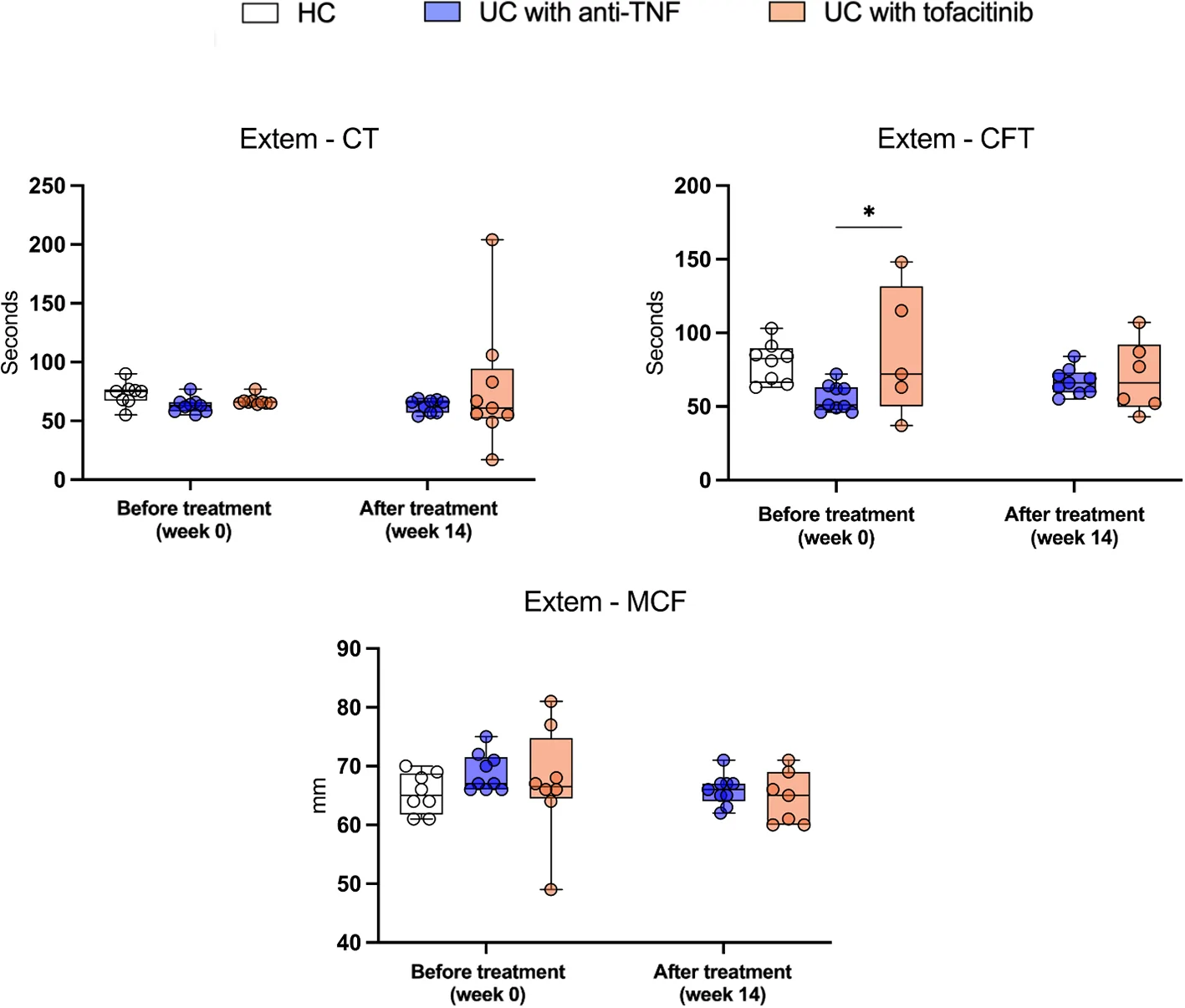
EXTEM coagulation parameters in patients with ulcerative colitis treated with anti-TNF or tofacitinib. Clotting time, clot formation time and maximum clot firmness parameters measured in the extrinsic activation with tissue factor (EXTEM) assay performed with whole blood obtained from healthy controls, patients before and after 14 weeks of treatment with anti-TNF or with tofacitinib. Patients were assessed at baseline and after 14 weeks of treatment, whereas HC were assessed at a single time point. Sample sizes varied according to data availability (n=5–9 per group). Longitudinal comparisons between anti-TNF- and tofacitinib-treated patients were performed using two-way repeated-measures ANOVA when complete paired observations were available or a mixed-effects model fitted by restricted maximum likelihood when repeated measurements were missing. Time, treatment, and time × treatment interaction effects were evaluated. Prespecified *post-hoc* comparisons were performed using Šídák’s multiple-comparisons test. Data are presented as mean ± SD. *p<0.05. CFT, clot formation time; CT, clotting time; HC, healthy control; MCF, maximum clot firmness; SD, standard deviation; TNF, tumor necrosis factor; UC, ulcerative colitis.

Whole blood from patients with UC and HC was analyzed using rotational thromboelastometry to assess coagulation following extrinsic and intrinsic pathway activation. Longitudinal analyses comparing patients receiving anti-TNF or tofacitinib showed no significant time-by-treatment interactions for EXTEM CT or MCF (p=0.548 and p=0.641, respectively). A significant time-by-treatment interaction was observed for EXTEM CFT (p=0.015; 95% CI, −50.97 to −6.68). However, CFT already differed significantly between treatment groups at baseline, before treatment initiation (95% CI, −60.47 to −3.74; adjusted p=0.025), whereas no significant between-group difference was observed at week 14 (95% CI, −30.70 to 24.15; adjusted p=0.951). Neither treatment group showed a significant within-group change in EXTEM CFT over time (**Figure 4**; **Supplementary Table 2**).

**Figure 5:**
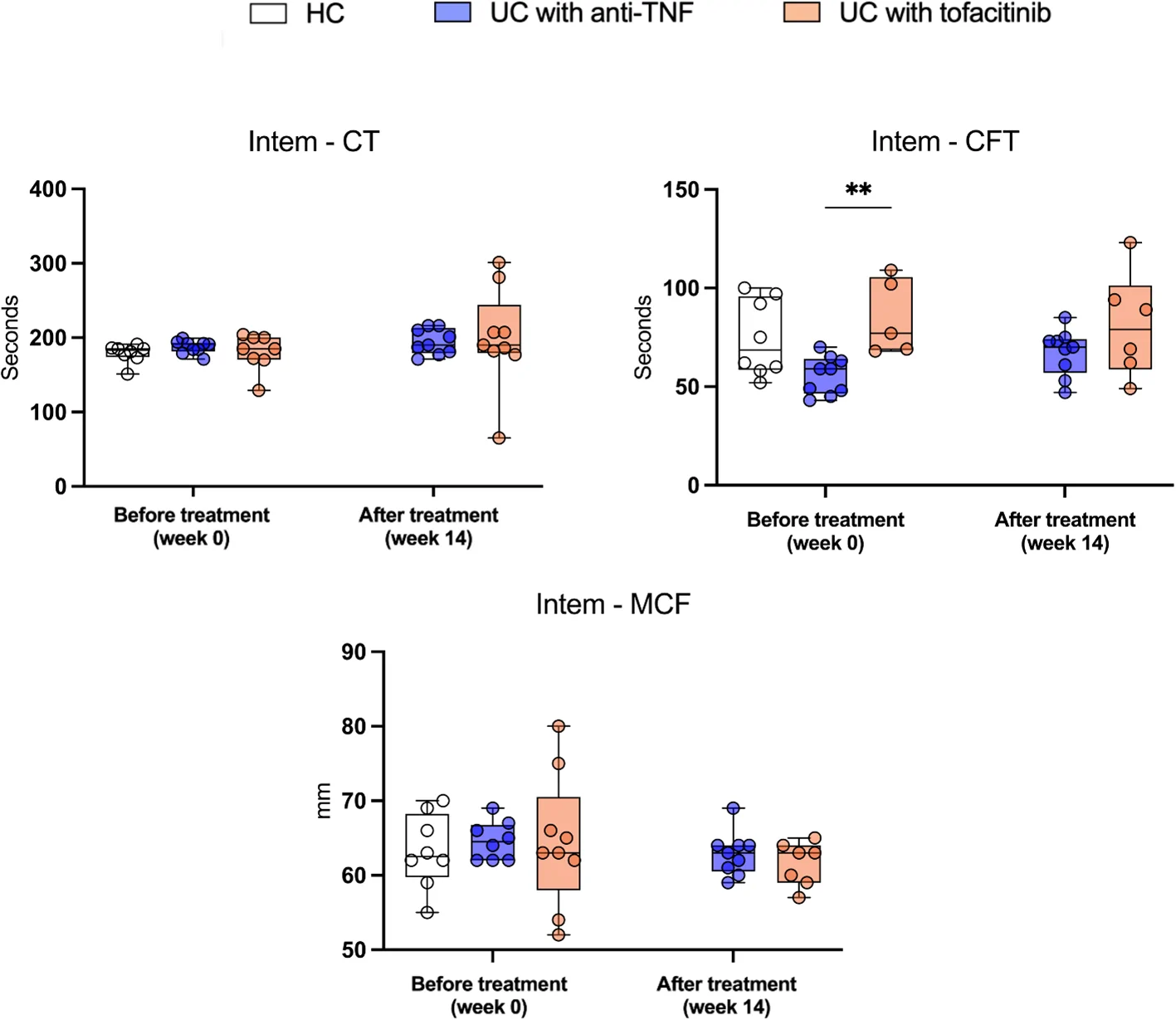
INTEM coagulation parameters in patients with ulcerative colitis treated with anti-TNF or tofacitinib. Clotting time, clot formation time and maximum clot firmness parameters measured in the intrinsic activation with tissue factor (INTEM) assay performed with whole blood obtained from healthy controls, patients treated with anti-TNF and patients treated with tofacitinib before and after 14 weeks of treatment. Patients were assessed at baseline and after 14 weeks of treatment, whereas HC were assessed at a single time point. Sample sizes varied according to data availability (n=5–9 per group). Longitudinal comparisons between anti-TNF- and tofacitinib-treated patients were performed using two-way repeated-measures ANOVA when complete paired observations were available or a mixed-effects model fitted by restricted maximum likelihood when repeated measurements were missing. Time, treatment, and time × treatment interaction effects were evaluated. Prespecified *post-hoc* comparisons were performed using Šídák’s multiple-comparisons test. Data are presented as mean ± SD. *p<0.05; **p<0.01. CFT, clot formation time; CT, clotting time; HC, healthy control; MCF, maximum clot firmness; SD, standard deviation; TNF, tumor necrosis factor; UC, ulcerative colitis.

Similarly, no significant time-by-treatment interactions were observed for INTEM CT or MCF (p=0.641 and p=0.991, respectively). For INTEM CFT, an overall treatment-group effect was observed (p=0.019), but there was no significant time-by-treatment interaction (p=0.120; 95% CI, −32.97 to 4.32). Importantly, INTEM CFT was significantly higher in patients subsequently receiving tofacitinib than in those receiving anti-TNF at baseline (95% CI, −49.54 to −6.45; adjusted p=0.010), before treatment initiation. This difference was no longer statistically significant at week 14 (95% CI, −34.37 to 7.04; adjusted p=0.241), and no significant longitudinal change was observed within the tofacitinib group (adjusted p=0.912) (**Figure 5**; **Supplementary Table 3**).

Separate cross-sectional comparisons with HC showed no significant differences in any of the EXTEM or INTEM parameters evaluated. Measured values for EXTEM and INTEM parameters are reported in **Supplementary Tables 4**, **5**.

## 4 Discussion

In this exploratory mechanistic study, tofacitinib treatment was not associated with a consistent prothrombotic pattern in platelet function or coagulation in patients with active UC when compared with anti-TNF therapy. Platelet aggregation at week 14 was lower in patients receiving tofacitinib than in those receiving anti-TNF therapy following stimulation with both ADP concentrations. However, a significant time-by-treatment interaction was observed only with 2.5 µM ADP, and no significant within-group change from baseline was observed in the tofacitinib group at either ADP concentration. Nevertheless, these findings should not be interpreted as evidence of an anti-aggregatory effect of tofacitinib, since within-group changes from baseline did not reach statistical significance and a significant time-by-treatment interaction was observed only at the lower ADP concentration. Flow cytometry did not show increased binding of PAC-1 or expression of CD62P or CD63 under basal conditions or after TRAP stimulation. Similarly, ROTEM^®^ analysis did not reveal a consistent pattern suggestive of accelerated clot formation or increased clot firmness associated with tofacitinib treatment. In addition, exploratory analyses did not identify significant sex-related differences in platelet activation or ROTEM^®^ parameters, and no significant associations with age remained after correction for multiple comparisons (**Supplementary Tables 6**, **7**).

These findings are clinically relevant because thromboembolic and cardiovascular safety concerns have been raised with tofacitinib, particularly after the ORAL Surveillance trial in rheumatoid arthritis (8, 12). In that study, the excess risk of major adverse cardiovascular events and venous thromboembolism was observed in a selected population of patients aged 50 years or older and with at least one additional cardiovascular risk factor. However, this population differs substantially from the typical UC population treated in clinical practice, and the extent to which those findings can be extrapolated to patients with UC remains uncertain.

Patients with inflammatory bowel disease have an intrinsically increased risk of venous and arterial thrombotic events, particularly during periods of active inflammation (11). Several mechanisms may contribute to this prothrombotic state, including platelet activation, thrombocytosis, endothelial dysfunction, activation of the coagulation cascade, and impaired fibrinolysis (13–16). In this context, it was reasonable to hypothesize that Janus kinase inhibition might further modify platelet function or coagulation dynamics and thereby contribute to thrombotic risk. However, our results do not support a direct proaggregant or platelet-activating effect of tofacitinib in patients with active UC.

The differences observed in clot formation time should be interpreted in the context of pre-existing between-group imbalances. For INTEM CFT, although an overall treatment-group effect was detected, there was no significant time-by-treatment interaction, and CFT was already significantly longer in patients subsequently receiving tofacitinib than in those receiving anti-TNF therapy at baseline, before treatment initiation. Moreover, this between-group difference was no longer statistically significant at week 14, and no significant longitudinal change was observed within the tofacitinib group. These findings indicate that the observed INTEM CFT difference primarily reflects a baseline imbalance rather than a treatment-emergent effect of tofacitinib. Similarly, the significant time-by-treatment interaction observed for EXTEM CFT occurred in the context of a significant baseline difference between treatment groups that was no longer present at week 14. Thus, although longitudinal trajectories differed for EXTEM CFT, this pattern does not indicate treatment-emergent acceleration of clot formation with tofacitinib. Together with the absence of significant longitudinal effects on CT or MCF, the overall ROTEM^®^ profile does not support increased coagulation potential associated with tofacitinib treatment.

Our findings are also consistent with clinical data from the UC development program of tofacitinib (17), in which venous thromboembolic events were uncommon and generally occurred in patients with additional thrombotic risk factors (18–20). They are also aligned with previous ex vivo data from our group showing that tofacitinib does not directly trigger platelet activation, platelet aggregation, or coagulation in samples from patients with active or quiescent UC (21). The present study extends those observations by evaluating patients receiving tofacitinib *in vivo* and by comparing them with both anti-TNF-treated patients and healthy controls.

Several limitations of our study should be acknowledged. First, the sample size was small, which limits statistical power and results in uncertainty around some effect estimates, as reflected by the width of several 95% confidence intervals. Accordingly, the present findings should be considered exploratory and should not be interpreted as demonstrating equivalence or excluding smaller treatment effects. Nevertheless, the longitudinal analyses did not identify a consistent directional pattern suggestive of increased platelet reactivity, platelet activation, accelerated clot formation, or increased clot firmness with tofacitinib. Indeed, the significant differences observed in platelet aggregation were in the direction of lower aggregation in the tofacitinib group, whereas differences in CFT were influenced by pre-existing baseline imbalances. Therefore, although small effects cannot be excluded, the absence of even a coherent prothrombotic trend argues against a major direct effect of tofacitinib on the platelet and ROTEM^®^-based coagulation endpoints assessed in this study. Second, the follow-up period was limited to 14 weeks, precluding assessment of longer-term hemostatic effects. However, this time frame covers the induction phase of tofacitinib therapy and is therefore suitable to explore early pharmacodynamic effects on platelet activation, platelet aggregation, and whole-blood clot formation. In the context of the UC clinical development program, deep vein thrombosis and pulmonary embolisms were not observed among tofacitinib-treated patients during the placebo-controlled induction studies, whereas thromboembolic events reported during tofacitinib exposure were uncommon and mainly occurred in longer-term extension cohorts and in patients with additional thrombotic risk factors (17). Third, healthy controls were significantly older than patients with UC. Since age may influence platelet reactivity and coagulation parameters, this imbalance limits the interpretation of direct comparisons between the patient groups and healthy controls. Nevertheless, healthy controls were primarily included as an external reference group, whereas the main assessment of the potential hemostatic effects of tofacitinib relied on comparisons with the anti-TNF group and on longitudinal changes from baseline. Greater interpretative weight should be placed on the comparisons between tofacitinib and anti-TNF therapy and on within-group longitudinal changes, while comparisons with healthy controls should be regarded as supportive contextual information. Fourth, the study focused on platelet aggregation, platelet activation markers, and ROTEM^®^-based coagulation parameters, but did not evaluate other potentially relevant mechanisms involved in thrombosis, such as endothelial activation, neutrophil extracellular trap formation, circulating microparticles, thrombin generation, or fibrinolytic pathways. Finally, this was a mechanistic study and was not designed to assess clinical thromboembolic outcomes.

Despite these limitations, the study has several strengths. It provides a prospective *in vivo* assessment of hemostatic parameters in patients with active UC treated with tofacitinib, incorporates complementary approaches to evaluate platelet aggregation, platelet activation, and whole-blood coagulation, and includes two clinically relevant comparator groups: patients treated with anti-TNF therapy and healthy controls. This design offers a more comprehensive assessment of the potential direct effects of tofacitinib on hemostasis than studies based exclusively on clinical event reporting.

In conclusion, in this exploratory mechanistic study, longitudinal assessment of platelet function and coagulation did not identify a consistent prothrombotic effect associated with tofacitinib treatment in patients with active UC. Although differences in ADP-induced platelet aggregation and clot formation time were detected, aggregation differences favored lower values with tofacitinib, whereas CFT findings were influenced by pre-existing baseline differences between treatment groups. These findings do not support increased platelet activation, aggregation, or coagulation as a major direct mechanism underlying the thromboembolic safety signal reported with tofacitinib. However, given the limited sample size and follow-up duration, these results should be interpreted as exploratory and require confirmation in larger longitudinal studies.

## Data Availability

The original contributions presented in the study are included in the article/**Supplementary Material**. Further inquiries can be directed to the corresponding author.

## References

[1] Xavier RJ Podolsky DK . Unravelling the pathogenesis of inflammatory bowel disease. Nature. (2007) 448:427–34. doi: 10.1038/nature06005 17653185

[2] Ramos GP Papadakis KA . Mechanisms of disease: Inflammatory bowel diseases. Mayo Clin Proc. (2019) 94:155–65. doi: 10.1016/j.mayocp.2018.09.013 30611442 PMC6386158

[3] Zhao J Lu Q Liu Y Shi Z Hu L Zeng Z . Th17 cells in inflammatory bowel disease: Cytokines, plasticity, and therapies. J Immunol Res. (2021) 2021:8816041. doi: 10.1155/2021/8816041 33553436 PMC7846404

[4] Baumgart DC Le Berre C . Newer biologic and small-molecule therapies for inflammatory bowel disease. N Engl J Med. (2021) 385:1302–15. doi: 10.1056/NEJMra1907607 34587387

[5] Sandborn WJ Su C Panes J . Tofacitinib as induction and maintenance therapy for ulcerative colitis. N Engl J Med. (2017) 377:496–7. doi: 10.1056/NEJMc1707500 28767341

[6] Gisbert JP Chaparro M . Janus kinase inhibitors for inflammatory bowel disease: Concise questions and answers on their use in clinical practice. Inflammation Bowel Dis. (2026) 32:775–802. doi: 10.1093/ibd/izaf273 41428330

[7] Gisbert JP Chaparro M . Janus kinase inhibitors in the management of acute severe ulcerative colitis: A comprehensive review. J Crohns Colitis. (2025) 19:jjaf021. doi: 10.1093/ecco-jcc/jjaf021 39886994

[8] Charles-Schoeman C Buch MH Dougados M Bhatt DL Giles JT Ytterberg SR . Risk of major adverse cardiovascular events with tofacitinib versus tumour necrosis factor inhibitors in patients with rheumatoid arthritis with or without a history of atherosclerotic cardiovascular disease: A post hoc analysis from ORAL Surveillance. Ann Rheum Dis. (2023) 82:119–29. doi: 10.1136/ard-2022-222259 36137735 PMC9811099

[9] Xeljanz, Xeljanz XR Tofacitinib: Drug safety communication - Due to an increased risk of blood clots and death with higher dose. FDA. Available online at: https://www.fda.gov/safety/medical-product-safety-information/xeljanz-xeljanz-xr-tofacitinib-drug-safety-communication-due-increased-risk-blood-clots-and-death (Accessed June 10, 2026).

[10] Tofacitinib (Xeljanz▼): new measures to minimise risk of venous thromboembolism and of serious and fatal infections. Available online at: https://www.gov.uk/drug-safety-update/tofacitinib-xeljanz-new-measures-to-minimise-risk-of-venous-thromboembolism-and-of-serious-and-fatal-infections (Accessed June 10, 2026).

[11] Danese S Motte Cd Cde L Fiocchi C . Platelets in inflammatory bowel disease: Clinical, pathogenic, and therapeutic implications. Am J Gastroenterol. (2004) 99:938–45. doi: 10.1111/j.1572-0241.2004.04129.x 15128364

[12] Kristensen LE Danese S Yndestad A Wang C Nagy E Modesto I . Identification of two tofacitinib subpopulations with different relative risk versus TNF inhibitors: An analysis of the open label, randomised controlled study ORAL Surveillance. Ann Rheum Dis. (2023) 82:901–10. doi: 10.1136/ard-2022-223715 36931693 PMC10314011

[13] Kim DS Moon W . Coagulopathy and platelet abnormalities in patients with inflammatory bowel disease. Korean J Intern Med. (2025) 40:866–75. doi: 10.3904/kjim.2025.118 41223869 PMC12611498

[14] Danese S de la Motte C Sturm A Vogel JD West GA Strong SA . Platelets trigger a CD40-dependent inflammatory response in the microvasculature of inflammatory bowel disease patients. Gastroenterology. (2003) 124:1249–64. doi: 10.1016/s0016-5085(03)00289-0 12730866

[15] Danese S Katz JA Saibeni S Papa A Gasbarrini A Vecchi M . Activated platelets are the source of elevated levels of soluble CD40 ligand in the circulation of inflammatory bowel disease patients. Gut. (2003) 52:1435–41. doi: 10.1136/gut.52.10.1435 12970136 PMC1773814

[16] Yoshida H Granger DN . Inflammatory bowel disease: A paradigm for the link between coagulation and inflammation. Inflammation Bowel Dis. (2009) 15:1245–55. doi: 10.1002/ibd.20896 19253306 PMC2713811

[17] Sandborn WJ Panés J Sands BE Reinisch W Su C Lawendy N . Venous thromboembolic events in the tofacitinib ulcerative colitis clinical development programme. Aliment Pharmacol Ther. (2019) 50:1068–76. doi: 10.1111/apt.15514 31599001 PMC6899755

[18] Chaparro M Garre A Mesonero F Rodríguez C Barreiro-de Acosta M Martínez-Cadilla J . Tofacitinib in ulcerative colitis: Real-world evidence from the ENEIDA Registry. J Crohns Colitis. (2021) 15:35–42. doi: 10.1093/ecco-jcc/jjaa145 32969471

[19] Taxonera C Olivares D Alba C . Real-world effectiveness and safety of tofacitinib in patients with ulcerative colitis: Systematic review with meta-analysis. Inflammation Bowel Dis. (2022) 28:32–40. doi: 10.1093/ibd/izab011 33586766

[20] Taneja V El-Dallal M Haq Z Tripathi K Systrom HK Wang LF . Effectiveness and safety of tofacitinib for ulcerative colitis: Systematic review and meta-analysis. J Clin Gastroenterol. (2022) 56:e323–33. doi: 10.1097/MCG.0000000000001608 34516458

[21] Sánchez-Sánchez C Suarez-Trujillo F Ramírez C Soleto I Mercado J Orejudo M . Effect of tofacitinib on hemostasis in patients with ulcerative colitis: A comparative ex vivo study. Pharm (Basel). (2025) 18:557. doi: 10.3390/ph18040557 40283992 PMC12030485

