# Supplementary material for: Tofacitinib does not affect platelet function or coagulation in patients with ulcerative colitis
Source: Front Immunol. 2026 Sep 10;17:1941163. doi: 10.3389/fimmu.2026.1941163 (PMC13601337; doi:10.3389/fimmu.2026.1941163)
Supplement: Supplementary file 1 [file Table1.docx]

Supplementary Material

# 1 Supplementary Figures and Tables

## 1.1 Supplementary Figures

**Supplementary Figure 1.** Representative flow cytometry gating strategy for the assessment of platelet activation markers under basal conditions and following stimulation with thrombin receptor-activating peptide. Platelets were initially identified based on their characteristic forward scatter (FSC) and side scatter (SSC) properties, and doublets were excluded by FSC-A versus FSC-H gating. Platelet identity was confirmed by the expression of CD41. Activated platelets were subsequently quantified by measuring the expression of PAC-1 as well as the degranulation markers CD62P (P-selectin) and CD63. Representative dot plots show the percentage of positive events for each marker under unstimulated conditions and after TRAP stimulation. Values within each gate indicate the percentage of positive events relative to the parent population. CD: cluster of differentiation; TRAP: thrombin receptor activating peptide.

**1.2 Supplementary Tables**

**Supplementary Table 1. Longitudinal analysis of ADP-induced platelet aggregation in patients with ulcerative colitis treated with anti-TNF agents or tofacitinib.** Platelet aggregation was evaluated following stimulation with 2.5 or 5 µM ADP. Longitudinal effects were assessed using mixed-effects models fitted by restricted maximum likelihood, with time (baseline and week 14), treatment group (anti-TNF and tofacitinib), and their interaction as fixed effects. The time × treatment interaction evaluates whether the change from baseline to week 14 differed between treatment groups. The interaction estimate represents the difference in longitudinal changes between groups and is reported with its 95% confidence interval (CI). Prespecified within-treatment comparisons between baseline and week 14 and between-treatment comparisons at each time point were performed using Šídák's multiple-comparisons test. Mean differences, 95% CIs, and adjusted p values are reported. Statistically significant results are shown in bold.

| **Repeated-measures analysis** | | | | |
| --- | --- | --- | --- | --- |
| **ADP concentration** | **Time, p** | **Treatment, p** | **Time × Treatment, p** | **Interaction estimate  (95% CI)** |
| **2.5 µM** | 0.412 | 0.085 | **0.045 *** | **−28.71 (−56.73 to −0.70)** |
| **5 µM** | 0.544 | **0.024 *** | 0.224 | −10.52 (−28.11 to 7.07) |
| **Within-treatment longitudinal comparisons** | | | | |
| **ADP concentration** | **Treatment** | **Baseline − Week 14 mean difference** | **95% CI** | **Adjusted *p*** |
| **2.5 µM** | Anti-TNF | −8.77 | −32.21 to 14.67 | 0.605 |
| **2.5 µM** | Tofacitinib | 19.94 | −2.66 to 42.54 | 0.088 |
| **5 µM** | Anti-TNF | −2.68 | −17.38 to 12.03 | 0.885 |
| **5 µM** | Tofacitinib | 7.84 | −6.36 to 22.03 | 0.349 |
| **Between-treatment comparisons at each time point** | | | | |
| **ADP concentration** | **Time point** | **Anti-TNF − Tofacitinib mean difference** | **95% CI** | **Adjusted *p*** |
| **2.5 µM** | Baseline | 3.17 | −23.84 to 30.18 | 0.954 |
| **2.5 µM** | Week 14 | **31.88** | **4.23 to 59.53** | **0.021 *** |
| **5 µM** | Baseline | 8.86 | −7.51 to 25.22 | 0.383 |
| **5 µM** | Week 14 | **19.38** | **2.61 to 36.15** | **0.021 *** |

**Supplementary Table 2.** **Longitudinal analysis of EXTEM coagulation parameters in patients with ulcerative colitis treated with anti-TNF agents or tofacitinib.** Clotting time (CT), clot formation time (CFT), and maximum clot firmness (MCF) were evaluated at baseline and after 14 weeks of treatment. Longitudinal effects were assessed using two-way repeated-measures ANOVA for CT and mixed-effects models fitted by restricted maximum likelihood for CFT and MCF because of missing repeated measurements. Models included time (baseline and week 14), treatment group (anti-TNF and tofacitinib), and their interaction. The time × treatment interaction evaluates whether the change from baseline to week 14 differed between treatment groups. The interaction estimate represents the difference in longitudinal changes between groups and is reported with its 95% confidence interval (CI). Prespecified within-treatment comparisons between baseline and week 14 and between-treatment comparisons at each time point were performed using Šídák's multiple-comparisons test. Mean differences, 95% CIs, and adjusted p values are reported. Statistically significant results are shown in bold.

| **Repeated-measures analysis** | | | | |
| --- | --- | --- | --- | --- |
| **Parameter** | **Time, *p*** | **Treatment, *p*** | **Time × Treatment, *p*** | **Interaction estimate  (95% CI)** |
| **CT (s)** | 0.573 | 0.326 | 0.548 | 11.00 (−27.03 to 49.03) |
| **CFT (s)** | 0.528 | 0.118 | **0.015 *** | **−28.83 (−50.97 to −6.68)** |
| **MCF (mm)** | 0.117 | 0.695 | 0.641 | 1.33 (−4.66 to 7.33) |
| **Within-treatment longitudinal comparisons** | | | | |
| **Parameter** | **Treatment** | **Baseline − Week 14 mean difference** | **95% CI** | **Adjusted *p*** |
| **CT (s)** | Anti-TNF | 0.33 | −30.96 to 31.62 | >0.999 |
| **CT (s)** | Tofacitinib | −10.67 | −41.96 to 20.62 | 0.655 |
| **CFT (s)** | Anti-TNF | −11.11 | −26.77 to 4.54 | 0.181 |
| **CFT (s)** | Tofacitinib | 17.72 | −2.98 to 38.41 | 0.096 |
| **MCF (mm)** | Anti-TNF | 3.00 | −1.67 to 7.67 | 0.243 |
| **MCF (mm)** | Tofacitinib | 1.67 | −3.55 to 6.88 | 0.683 |
| **Between-treatment comparisons at each time point** | | | | |
| **Parameter** | **Time point** | **Anti-TNF − Tofacitinib mean difference** | **95% CI** | **Adjusted *p*** |
| **CT (s)** | Baseline | −3.67 | −33.56 to 26.22 | 0.950 |
| **CT (s)** | Week 14 | −14.67 | −44.56 to 15.22 | 0.450 |
| **CFT (s)** | Baseline | **−32.11** | **−60.47 to −3.74** | **0.025 *** |
| **CFT (s)** | Week 14 | −3.28 | −30.70 to 24.15 | 0.951 |
| **MCF (mm)** | Baseline | 1.64 | −4.88 to 8.16 | 0.804 |
| **MCF (mm)** | Week 14 | 0.30 | −6.39 to 7.00 | 0.993 |

CFT: clot formation time; CT: clotting time; MCF: maximum clot firmness; TNF: tumor necrosis factor.

**Supplementary Table 3. Longitudinal analysis of INTEM coagulation parameters in patients with ulcerative colitis treated with anti-TNF agents or tofacitinib.** Clotting time (CT), clot formation time (CFT), and maximum clot firmness (MCF) were evaluated at baseline and after 14 weeks of treatment. Longitudinal effects were assessed using mixed-effects models fitted by restricted maximum likelihood (REML) because of missing repeated measurements, with time (baseline and week 14), treatment group (anti-TNF and tofacitinib), and their interaction as fixed effects. The time × treatment interaction evaluates whether the change from baseline to week 14 differed between treatment groups. The interaction estimate represents the difference in longitudinal changes between groups and is reported with its 95% confidence interval (CI). Prespecified within-treatment comparisons between baseline and week 14 and between-treatment comparisons at each time point were performed using Šídák's multiple-comparisons test. Mean differences, 95% CIs, and adjusted p values are reported. Statistically significant results are shown in bold.

| **Repeated-measures analysis** | | | | |
| --- | --- | --- | --- | --- |
| **Parameter** | **Time, p** | **Treatment, p** | **Time × Treatment, p** | **Interaction estimate (95% CI)** |
| **CT (s)** | 0.314 | 0.954 | 0.641 | 11.93 (−39.70 to 63.56) |
| **CFT (s)** | 0.313 | **0.019 *** | 0.120 | −14.33 (−32.97 to 4.32) |
| **MCF (mm)** | 0.233 | 0.964 | 0.991 | 0.03 (−5.72 to 5.78) |
| **Within-treatment longitudinal comparisons** | | | | |
| **Parameter** | **Treatment** | **Baseline − Week 14 mean difference** | **95% CI** | **Adjusted *p*** |
| **CT (s)** | Anti-TNF | −7.00 | −48.42 to 34.42 | 0.906 |
| **CT (s)** | Tofacitinib | −18.93 | −61.63 to 23.76 | 0.518 |
| **CFT (s)** | Anti-TNF | −11.67 | −24.88 to 1.55 | 0.086 |
| **CFT (s)** | Tofacitinib | 2.66 | −14.74 to 20.06 | 0.912 |
| **MCF (mm)** | Anti-TNF | 1.68 | −2.95 to 6.30 | 0.611 |
| **MCF (mm)** | Tofacitinib | 1.65 | −3.23 to 6.52 | 0.651 |
| **Between-treatment comparisons at each time point** | | | | |
| **Parameter** | **Treatment** | **Baseline − Week 14 mean difference** | **95% CI** | **Adjusted *p*** |
| **CT (s)** | Anti-TNF | −7.00 | −48.42 to 34.42 | 0.906 |
| **CT (s)** | Tofacitinib | −18.93 | −61.63 to 23.76 | 0.518 |
| **CFT (s)** | Anti-TNF | −11.67 | −24.88 to 1.55 | 0.086 |
| **CFT (s)** | Tofacitinib | 2.66 | −14.74 to 20.06 | 0.912 |
| **MCF (mm)** | Anti-TNF | 1.68 | −2.95 to 6.30 | 0.611 |
| **MCF (mm)** | Tofacitinib | 1.65 | −3.23 to 6.52 | 0.651 |

CFT: clot formation time; CT: clotting time; MCF: maximum clot firmness; TNF: tumor necrosis factor.

**Supplementary Table 4.** Clot formation, clot formation time and maximum clot firmness parameters measured in the extrinsic activation with tissue factor (EXTEM) assay performed with whole blood obtained from healthy controls, patients treated with anti-TNF and patients treated with tofacitinib.

|  | **EXTEM assay** | | | | | | | | |
| --- | --- | --- | --- | --- | --- | --- | --- | --- | --- |
|  | **CT (seconds)** | | | **CFT (seconds)** | | | **MCF (mm)** | | |
|  | **HC** | **UC patients with anti-TNF** | **UC patients with tofacitinib** | **HC** | **UC patients with anti-TNF** | **UC patients withto facitinib** | **HC** | **UC patients with anti-TNF** | **UC patients with tofacitinib** |
| **Before treatment (week 0), mean (SD)** | 72.9 (10) | 63.2 (6) | 66.9 (4) | 80.1 (14) | 55.8 (9) | 87.0 (44) | 65.4 (3) | 68.9 (3) | 67.2 (9) |
| **After treatment (week 14), mean (SD)** | - | 62.9 (6) | 80.2 (56) | - | 66.9 (9) | 68.8 (27) | - | 65.9 (3) | 64.3 (5) |

CFT: clot formation time; CT: clotting time; HC: healthy controls; MCF: maximum clot firmness; SD: standard deviation; TNF: tumor necrosis factor; UC: ulcerative colitis.

**Supplementary Table 5.** Clot formation, clot formation time and maximum clot firmness parameters measured in the intrinsic activation with tissue factor (INTEM) assay performed with whole blood obtained from healthy controls, patients treated with anti-TNF and patients treated with tofacitinib.

|  | **INTEM assay** | | | | | | | | | |
| --- | --- | --- | --- | --- | --- | --- | --- | --- | --- | --- |
|  | **CT (seconds)** | | | **CFT (seconds)** | | | **MCF (mm)** | | | |
|  | **HC** | **UC patients with anti-TNF** | **UC patients with tofacitinib** | **HC** | **UC patients with anti-TNF** | **UC patients with tofacitinib** | **HC** | **UC patients with anti-TNF** | **UC patients with tofacitinib** |  |
| **Before treatment (week 0), mean (SD)** | 178.6 (12) | 187.3 (9) | 180.6 (24) | 74.5 (19) | 55.7 (10) | 85.0 (19) | 63.2 (5) | 64.6 (3) | 64.4 (9) |  |
| **After treatment (week 14), mean (SD)** | - | 194.0 (17) | 201.0 (72) | - | 67.3 (12) | 83.4 (29) | - | 62.9 (3) | 61.0 (3) |  |

CFT: clot formation time; CT: clotting time; HC: healthy controls; MCF: maximum clot firmness; SD: standard deviation; TNF: tumor necrosis factor; UC: ulcerative colitis.

**Supplementary Table 6. Sex-based comparisons of platelet activation and coagulation parameters.** Platelet activation parameters, including PAC-1 binding and CD62P and CD63 expression under unstimulated and TRAP-stimulated conditions, and coagulation parameters assessed by ROTEM® (EXTEM and INTEM) were compared between male and female patients at baseline and after 14 weeks of treatment. Comparisons were performed using two-tailed Mann–Whitney U tests. A p-value <0.05 was considered statistically significant. CT, clotting time; CFT, clot formation time; MCF, maximum clot firmness; TRAP, thrombin receptor-activating peptide.

| **Assessment** | **Time point** | **Condition / Parameter** | **Male vs Female, Mann–Whitney *p*** |
| --- | --- | --- | --- |
| **Platelet activation** | Baseline | PAC-1, unstimulated | 0.8182 |
|  |  | CD62P, unstimulated | 0.1797 |
|  |  | CD63, unstimulated | 0.5887 |
|  |  | PAC-1, TRAP-stimulated | 0.5887 |
|  |  | CD62P, TRAP-stimulated | 0.9372 |
|  |  | CD63, TRAP-stimulated | 0.8182 |
|  | Week 14 | PAC-1, unstimulated | 0.5303 |
|  |  | CD62P, unstimulated | 0.5303 |
|  |  | CD63, unstimulated | >0.9999 |
|  |  | PAC-1, TRAP-stimulated | 0.2677 |
|  |  | CD62P, TRAP-stimulated | 0.6755 |
|  |  | CD63, TRAP-stimulated | 0.6389 |
| **ROTEM®** | Baseline | EXTEM CT | 0.0900 |
|  |  | EXTEM CFT | 0.1442 |
|  |  | EXTEM MCF | 0.3733 |
|  |  | INTEM CT | 0.2443 |
|  |  | INTEM CFT | 0.6863 |
|  |  | INTEM MCF | 0.3616 |
|  | Week 14 | EXTEM CT | 0.1060 |
|  |  | EXTEM CFT | 0.1332 |
|  |  | EXTEM MCF | 0.1451 |
|  |  | INTEM CT | 0.7787 |
|  |  | INTEM CFT | 0.3357 |
|  |  | INTEM MCF | 0.2379 |

**Supplementary Table 7. Associations between age and platelet activation and coagulation parameters.** Associations between patient age and platelet activation parameters, including PAC-1 binding and CD62P and CD63 expression under unstimulated and TRAP-stimulated conditions, and coagulation parameters assessed by ROTEM® (EXTEM and INTEM) were evaluated at baseline and after 14 weeks of treatment using two-tailed Spearman rank correlations. Results are reported as Spearman's correlation coefficient (ρ) and p-value. p-values were adjusted for multiple comparisons using the Benjamini–Hochberg false discovery rate procedure. CT, clotting time; CFT, clot formation time; MCF, maximum clot firmness; TRAP, thrombin receptor-activating peptide.

| **Parameter** | **Time point** | **Condition** | **Spearman ρ** | **Adjusted p value** |
| --- | --- | --- | --- | --- |
| PAC-1 | Baseline | Unstimulated | 0.158 | 0.6230 |
| CD62P |  |  | 0.371 | 0.2334 |
| CD63 |  |  | 0.063 | 0.8467 |
| PAC-1 |  | TRAP | −0.228 | 0.4740 |
| CD62P |  |  | 0.785 | 0.0036 |
| CD63 |  |  | 0.077 | 0.8124 |
| PAC-1 | Week 14 | Unstimulated | 0.126 | 0.6947 |
| CD62P |  |  | −0.028 | 0.9337 |
| CD63 |  |  | −0.032 | 0.9258 |
| PAC-1 |  | TRAP | −0.070 | 0.8294 |
| CD62P |  |  | 0.511 | 0.0916 |
| CD63 |  |  | −0.273 | 0.3870 |
| EXTEM CT | Baseline | — | 0.047 | 0.8470 |
| EXTEM CFT |  |  | −0.136 | 0.6397 |
| EXTEM MCF |  |  | 0.144 | 0.5925 |
| INTEM CT |  |  | −0.162 | 0.5314 |
| INTEM CFT |  |  | 0.250 | 0.3840 |
| INTEM MCF |  |  | 0.238 | 0.3708 |
| EXTEM CT | Week 14 |  | 0.005 | 0.9855 |
| EXTEM CFT |  |  | 0.182 | 0.5293 |
| EXTEM MCF |  |  | −0.187 | 0.5011 |
| INTEM CT |  |  | −0.210 | 0.4318 |
| INTEM CFT |  |  | 0.088 | 0.7550 |
| INTEM MCF |  |  | −0.160 | 0.5657 |
